# Identification of key mRNAs and signaling pathways in obsessive compulsive disorder based on weighted gene co‐expression network analysis and cytoHubba plugin

**DOI:** 10.1002/brb3.3412

**Published:** 2024-04-25

**Authors:** Xiaoming Zhang, Yanru Liu, Bin Guo, Binbin Li, Huaqing Liu, Zhiren Wang

**Affiliations:** ^1^ Department of Psychiatry Beijing Huilongguan Hospital Beijing China

**Keywords:** DEmRNAs, functional enrichment, immune cells, obsessive‐compulsive disorder, weighted gene co‐expression network analysis

## Abstract

**Purpose:**

Obsessive‐compulsive disorder (OCD) is a complex psychiatric disorder. Genetic and broad environmental factors are common risk factors for OCD. The purpose of this study is to explore the molecular mechanism of OCD and to find new molecular targets for the diagnosis and management of OCD.

**Methods:**

All data were downloaded from public dataset. Key modules and candidate key mRNAs were identified based on weighted gene co‐expression network analysis (WGCNA). The “limma” R package was used for differential expression analysis of mRNAs. Subsequently, functional enrichment analysis of differentially expressed mRNAs (DEmRNAs) was also carried out. In addition, a diagnostic model was constructed. Finally, the infiltration level of immune cells in OCD and its correlation with multicentric key DEmRNAs were analyzed.

**Results:**

Green and red modules were selected as the hub modules. A total of 447 mRNAs were considered candidate key mRNAs according to GS > 0.2 and MM > 0.3. A total of 26 DEmRNAs in the same direction were identified in the GSE60190 and GSE78104 datasets. A total of 26 DEmRNAs were intersected with candidate key mRNAs in WGCNA to obtain 10 intersection DEmRNAs (HSPB1, ITPK1, CBX7, PPP1R10, TAOK1, PISD, MKNK2, RWDD1, PPA1, and RELN). However, only four DEmRNAs (HSPB1, TAOK1, MKNK2, and PPA1) predicted related drugs. Subsequently, receiver operating characteristic analysis shows that the diagnostic model has high diagnostic value. Moreover, six multicentric key DEmRNAs (SNRPF, SNRNP70, PRPF8, NOP56, EPRS, and CCT2) were screened by UpSet package. Finally, six multicentric key DEmRNAs were found to be associated with immune cells.

**Conclusion:**

The key molecules obtained in this study lay a foundation for further research on the molecular mechanism of OCD.

## Graphical abstract

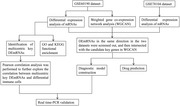

## 1 INTRODUCTION

Obsessive‐compulsive disorder (OCD) is a complex psychiatric disorder characterized by recurring intrusive and disturbing thoughts (obsessions) and repetitive behaviors (compulsions) that cause significant distress and impairment (Goodman et al., 2014). Genetic and broad environmental factors are common risk factors for OCD (Stein et al., 2019). OCD occurs mostly in adolescence and early adulthood (Mahjani et al., 2021). In addition, peoples with OCD have significantly higher risk of death from natural or non‐natural causes than the general population (Meier et al., 2016). The common treatment for OCD is psychotherapy, drug therapy, and brain regulation (Drubach, 2015). Previous studies have shown that OCD is regulated by various molecules, such as phosphodiesterase 4D (PDE4D) (Huang et al., 2019), 5‐HT_2C_ receptor (Hatakama et al., 2022), and A‐kinase anchoring protein 13 (AKAP13) (Su et al., 2018). In addition, immune processes may be associated with OCD (Marazziti et al., 2018). The proportion of T‐regulatory cells was significantly lower in OCD patients compared with healthy controls (Subbanna et al., 2021). Although great progress has been made in understanding the pathogenesis of OCD, the specific mechanisms remain elusive. Therefore, the continuous exploration of the molecular mechanism of OCD to find new targets is helpful for the diagnosis and treatment of patients.

Weighted gene co‐expression network analysis (WGCNA) can find highly correlated clusters (modules) based on microarray and sequencing data (Langfelder & Horvath, 2008). Correlation networks facilitate the screening of genes that can be used to identify candidate biomarkers or therapeutic targets (Langfelder & Horvath, 2008). CytoHubba plugin provides 11 topological analysis methods and 6 centralities based on shortest paths (Chin et al., 2014). The CytoHubba plugin can retrieve subnetworks from the entire large protein–protein interaction (PPI) set and evaluate node importance on selected subnetworks (Chin et al., 2014). Therefore, key molecules identified by WGCNA and cytoHubba plugin have important potential research value for disease. In this study, the mRNA data were obtained from the Gene Expression Omnibus (GEO, https://www.ncbi.nlm.nih.gov/geo/) database. The differential expression and functional enrichment of OCD mRNAs was analyzed. Potential key molecular markers in OCD were also screened based on WGCNA and cytoHubba plugin. Moreover, diagnostic models have also been constructed to aid clinical diagnosis. The key molecules obtained in this study lay a foundation for further research on the molecular mechanism of OCD. In addition, the constructed diagnostic model has high diagnostic value and may contribute to the management of OCD.

## 2 MATERIALS AND METHODS

### 2.1 Sources of data

The mRNA data were obtained from the GEO database. The “obsessive‐compulsive disorder” and “*Homo sapiens*” are used as keywords to search in the GEO database. Subsequently, cell line or animal level studies and single‐sample studies were excluded. Finally, two datasets GSE60190 (16 patients and 102 normal controls) and GSE78104 (30 patients and 30 normal controls) were included in the study. The GSE60190 and GSE78104 datasets were derived from tissue samples and blood samples, respectively. Probe‐level data were converted to gene expression values using GPL annotation files. Multiple probes correspond to the same gene and take the average value. The GSE60190 dataset was used as discovery array, and the GSE78104 dataset was used as verification array.

### 2.2 Analysis of weighted gene co‐expression network

The “WGCNA” R package was used to analyze all mRNAs in the GSE60190 dataset to construct a scale‐free gene co‐expression network. First, the “hclust” function was used to cluster the sample data to detect outliers. Then, the “pickSoftThreshold” function was used to select an appropriate soft‐threshold power regulator to construct a scale‐free topology (height 0.90, *β* value 9). According to the kernel value calculate the adjacency matrix. The adjacency matrix was transformed into topological overlap matrix (TOM) and corresponding dissimilarity matrix (1‐TOM). Subsequently, similar genes were clustered together based on gene expression patterns. Modules were divided according to the function of “cutreeDynamic” with default parameters. As the modules identified by the dynamic tree cutting algorithm may be similar, they are combined with a truncation of 0.25 height (Zhong et al., 2019).

### 2.3 Identification of hub modules and candidate key mRNAs

The “moduleEigengenes” function was used to calculate the module eigengene of each module (Zhong et al., 2019). The “Pearson” method was used to analyze the correlation between module eigengene and OCD. Subsequently, the two modules with the highest positive and negative correlation with OCD were selected as hub modules. Candidate key mRNAs were selected based on module connectivity (MM) and clinical trait relationships (GS) of each gene in the hub module (Lin et al., 2020). The screening criteria were GS > 0.2 and MM > 0.3.

### 2.4 Identification and functional enrichment of differentially expressed mRNAs (DEmRNAs)

The “limma” R package was used for differential expression analysis of mRNAs. False discovery rate (FDR) < 0.05 and |log2fold change| (|log2FC|) > 0.2 were the screening criteria for differentially expressed mRNAs (DEmRNAs) in GSE60190 dataset. *p* < .05 was the screening criteria for DEmRNAs in GSE78104 dataset. David was used for Gene Ontology (GO) and Kyoto Encyclopedia of Genes and Genomes (KEGG) functional enrichment analysis of DEmRNAs in the GSE60190 dataset to identify potential biological pathways of OCD. GO functional annotations are divided into biological processes (BP), cellular components (CC), and molecular functions (MF), so as to define and describe the functions of genes in many aspects. *p* < .05 was considered significant difference.

### 2.5 Drug prediction of OCD

DEmRNAs in the same direction in the two datasets were screened out and then intersected with the candidate key genes in WGCAN. Drugs related to the intersection DEmRNAs were screened out based on the DGIdb database (https://dgidb.org/). Hope to provide a new perspective for the diagnosis, treatment, and research of OCD.

### 2.6 Construction of diagnostic model

To facilitate clinical application, the Lasso method was used to screen the intersection DEmRNAs to reduce the number of DEmRNAs in the diagnostic model. Subsequently, multivariate cox regression was used to construct a risk score model for OCD. The calculation formula was as follows: Riskscore=∑Coef(i)×Exp(i). The receiver operating characteristic (ROC) analysis was performed by using pROC package in R language. Area under the curve (AUC) was used to evaluate the accuracy of the diagnostic model. AUC > 0.7 represent a high diagnostic accuracy of the model (Šimundić, 2009).

### 2.7 Identification of multicentric key DEmRNAs

The DEmRNAs in the GSE60190 dataset were input into the STRING database (https://cn.string‐db.org/) to construct a PPI network. Cytoscape was used to visualize the PPI network. Subsequently, the PPI network was screened using the cytoHubba plugin in Cytoscape. Five groups of central DEmRNAs were screened out by five algorithms (degree, maximum neighborhood component, maximal clique centrality, edge percolated component, and betweenness) in cytoHubba plug‐in. The UpSet package was used to process the top 20 central DEmRNAs of each algorithm and screen out the common DEmRNAs as the multicentric key DEmRNAs.

### 2.8 Analysis of immune cells infiltration levels

Gene sets marking each immune cell type were obtained from the Charoentong study (Charoentong et al., 2017). Single‐sample gene set enrichment analysis algorithm was used to quantify the relative abundance of each immune cell in the immune microenvironment (IME) of OCD. Wilcoxon test was used to statistically analyze the significance of differences between OCD and normal control groups. Subsequently, the correlation between immune cells and multicentric key genes was analyzed.

### 2.9 Real‐time‐polymerase chain reaction (real‐time‐PCR) validation

The detailed inclusion criteria for patients with OCD were as follows: (1) Patients met the DSM‐IV diagnostic criteria for OCD; (2) the total severity score of the patient's Yale Brown Obsessive Compulsive Disorder Scale is ≥16; (3) patients were between 16 and 54 years old and had at least primary education; and (4) patients had never been exposed to or received any psychotropic medication or had not taken any psychotropic medication for at least 8 weeks prior to the assessment. Patients who had suicidal attempt, were pregnant or lactating, and were in poor health were excluded. Individuals in the control group were normal healthy people who underwent routine health checkups, aged 16–54 years, and had at least primary education. According to the above screening criteria, blood samples from nine patients with OCD and nine healthy controls were included in this study. Total RNA was extracted from blood samples using the RNAliquid overdrive whole blood (liquid sample) total RNA extraction kit. Subsequently, reverse transcription and real‐time‐PCR were performed using FastKing cDNA first‐strand synthesis kit and SuperReal PreMix Plus (SYBR Green), respectively. Gene‐9660 fluorescence quantitative PCR instrument was used for detection. The relative expression levels were calculated by 2^−△△^ *^CT^* method (Livak & Schmittgen, 2001).

### 2.10 Statistical analysis

All statistics were performed using R software. The “limma” R package was used for differential expression analysis of mRNAs. The Wilcoxon test was used to statistically analyze the significance of differences between OCD and normal control groups. Pearson correlation analysis was performed to further explore the correlation between multicentric key DEmRNAs and differential immune cells in OCD. For the real‐time‐PCR, *t*‐test was used to evaluate the statistical significance.

## 3 RESULTS

### 3.1 WGCNA

**FIGURE 1:**
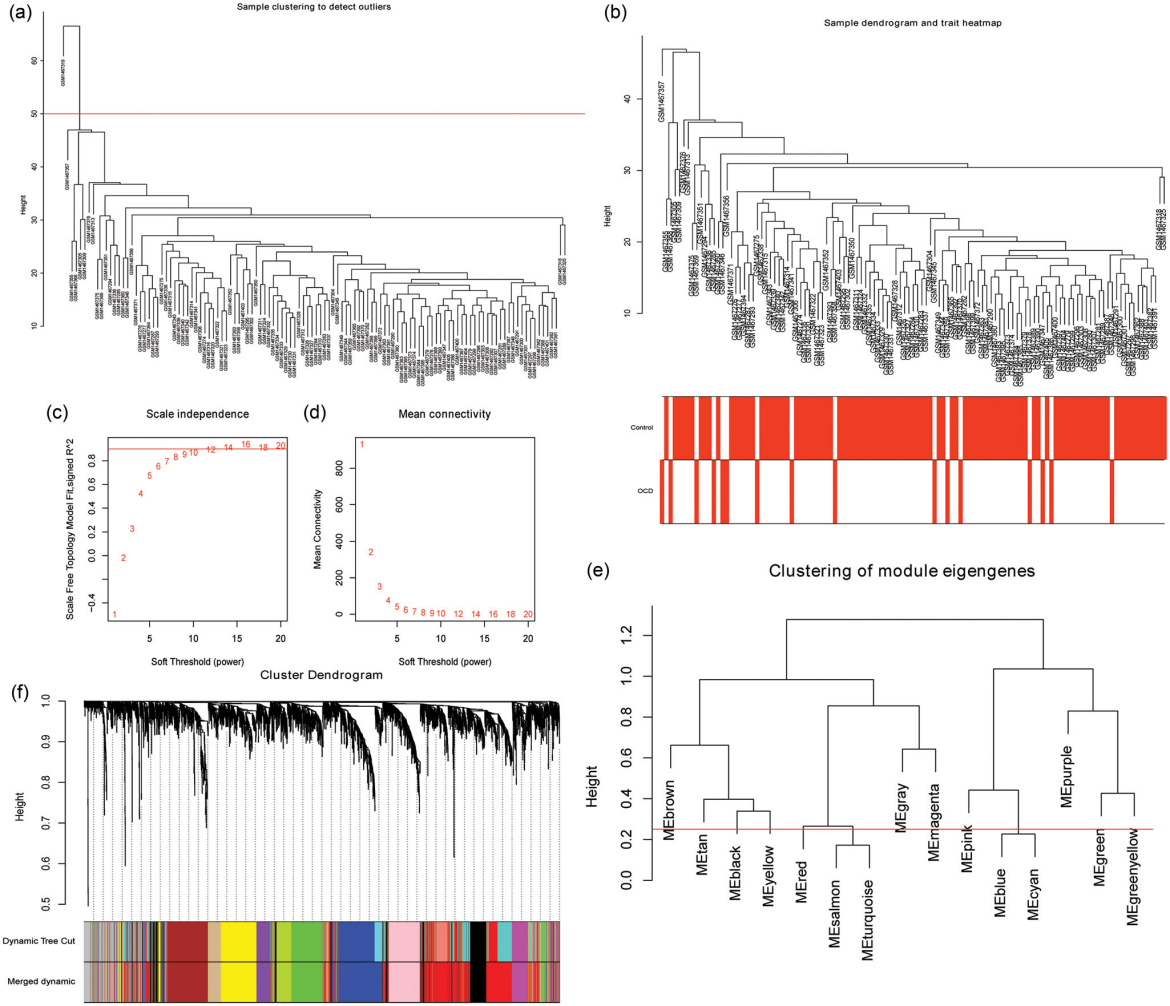
Construction of weighted gene co‐expression network: (a) sample clustering dendrogram to detect outliers; (b) sample dendrogram and trait heat map; (c) scale‐free fit index of different soft‐threshold powers (*β*); (d) mean connectivity of various soft‐threshold powers; (e) clustering dendrogram of module eigenelements; and (f) module clustering dendrogram. Different colors represent different modules.

To identify genes associated with OCD, WGCNA was used to analyze genes in the top 25% of variance from 118 samples. Cluster the samples to remove abnormal samples (GSM1467319) (Figure 1a,b). To build a scale‐free network, we picked *β* = 9 as the soft‐thresholding power (Figure 1c,d). A clustering tree was constructed by setting the minimum number of genes in modules to 50. A total of 14 modules were separated (gray modules are not counted). Subsequently, the dynamic cutting tree method was used to merge the modules whose dissimilarity <25%. Finally, 11 modules are determined (gray modules are not counted) (Figure 1e,f).

### 3.2 Hub modules and candidate key mRNAs

**FIGURE 2:**
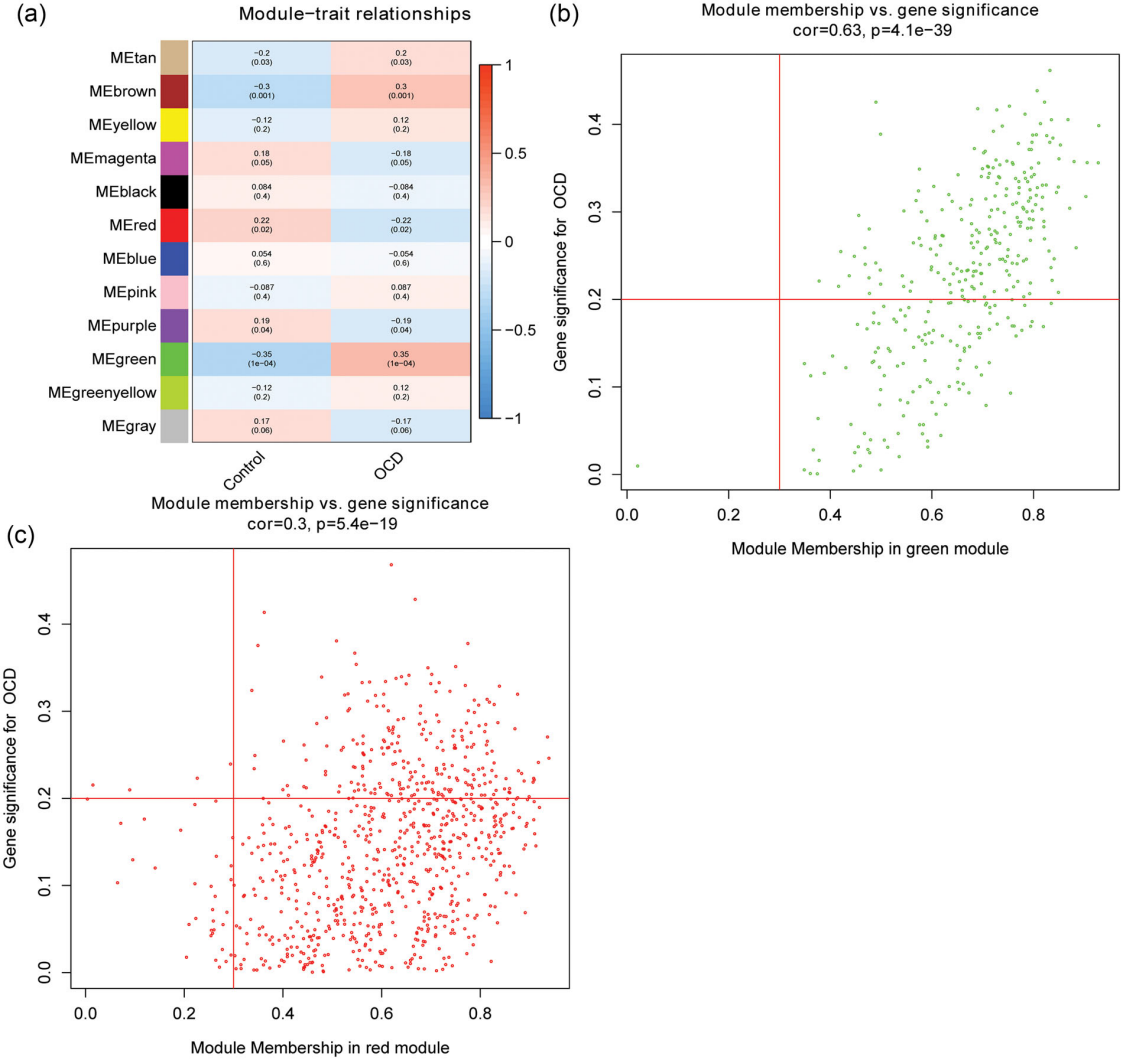
Identification of hub module and candidate key mRNAs: (a) heat map of the correlation between ME and OCD; (b) scatter plot of candidate key mRNAs screening in green modules; and (c) scatter plot of candidate key mRNAs screening in red modules.

Pearson correlation analysis showed that the green module had the highest positive correlation with OCD, whereas the red module had the highest negative correlation with OCD (Figure 2a). Therefore, the green and red modules were selected as the hub modules. The mRNAs in the modules were screened according to GS > 0.2 and MM > 0.3. It was found that there were 214 mRNAs remaining in the green module and 233 mRNAs remaining in the red module (Figure 2b,c).A total of 447 mRNAs were considered candidate key mRNAs.

### 3.3 Identification and functional enrichment of DEmRNAs in GSE60190 dataset

**FIGURE 3:**
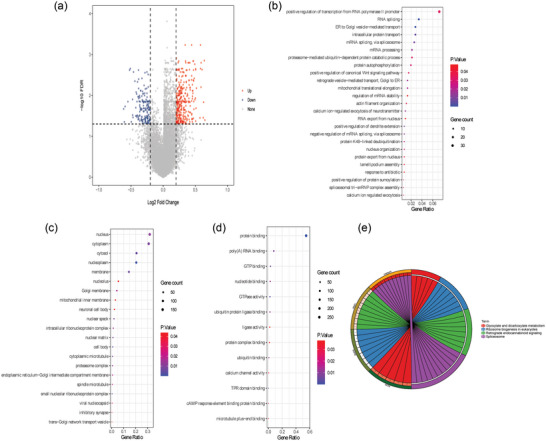
Identification and functional enrichment analysis of DEmRNAs: (a) volcano plot of DEmRNAs in the GSE60190 dataset; (b) BP term in GO functional enrichment of DEmRNAs; (c) CC term in GO functional enrichment of DEmRNAs; (d) MF term in GO functional enrichment of DEmRNAs; and (e) KEGG functional enrichment of DEmRNAs.

Differential expression of mRNA in GSE60190 dataset (remove GSM1467319 sample) was analyzed according to FDR < 0.05 and |log2FC| > 0.2. Compared with normal control group, 496 DEmRNAs were identified in the OCD group. Among them, 324 DEmRNAs were upregulated, and 172 DEmRNAs were downregulated (Figure 3a). GO functional analysis found that in the GO:BP term, DEmRNAs were mainly enriched in positive regulation of transcription from RNA polymerase II promoter and RNA splicing (Figure 3b). In the GO:CC term, DEmRNAs were mainly enriched in nucleus and cytoplasm (Figure 3c). In the GO:MF term, DEmRNAs were mainly enriched in protein binding and poly(A) RNA binding (Figure 3d). Only four pathways spliceosome, retrograde endocannabinoid signaling, ribosome biogenesis in eukaryotes, and glyoxylate and dicarboxylate metabolism were enriched in KEGG (Figure 3e).

### 3.4 Drug prediction of OCD

**FIGURE 4:**
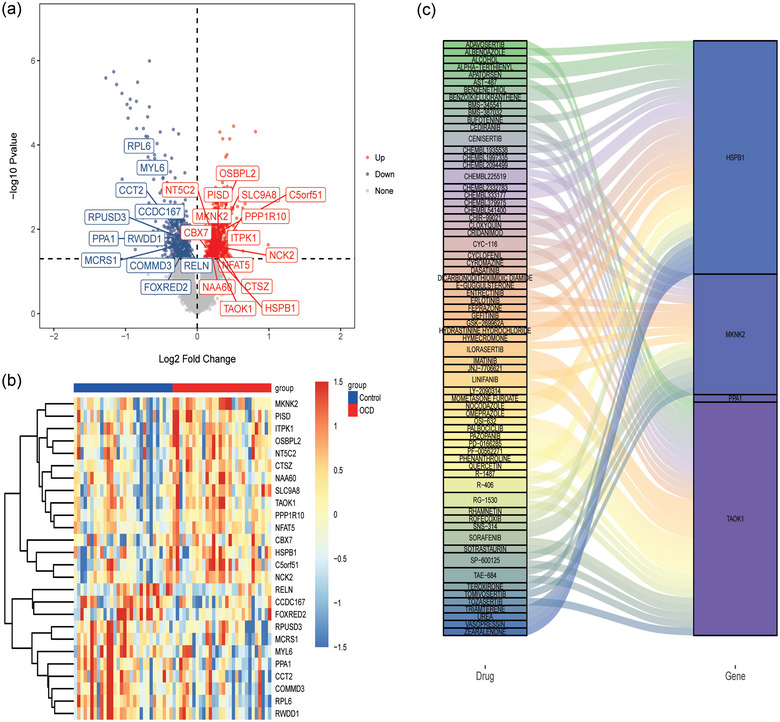
Drug prediction. (a) Volcano plot of DEmRNAs in the GSE78104 dataset. The DEmRNAs marked with names are the same direction DEmRNAs of the GSE60190 and GSE78104 datasets. (b) Heat maps of 26 DEmRNAs; and (c) correspondence map of intersection DEmRNAs and related drugs.

A total of 26 DEmRNAs in the same direction were identified in the GSE60190 and GSE78104 datasets (Figure 4a,b). A total of 26 DEmRNAs were intersected with candidate key mRNAs in WGCNA to obtain 10 intersection DEmRNAs (heat shock protein family B (small) member 1 [HSPB1], inositol‐tetrakiphosphate 1‐kinase [ITPK1], chromobox 7 [CBX7], protein phosphatase 1 regulatory subunit 10 [PPP1R10], altered TAO kinase 1 [TAOK1], phosphatidylserine decarboxylase [PISD], MAPK interacting serine/threonine kinase 2 [MKNK2], RWD domain containing 1 [RWDD1], pyrophosphatase 1 [PPA1], and reelin [RELN]). Subsequently, drug prediction was performed on 10 DEmRNAs. However, only four DEmRNAs (HSPB1, TAOK1, MKNK2, and PPA1) predicted related drugs (Figure 4c).

### 3.5 Diagnostic model

**FIGURE 5:**
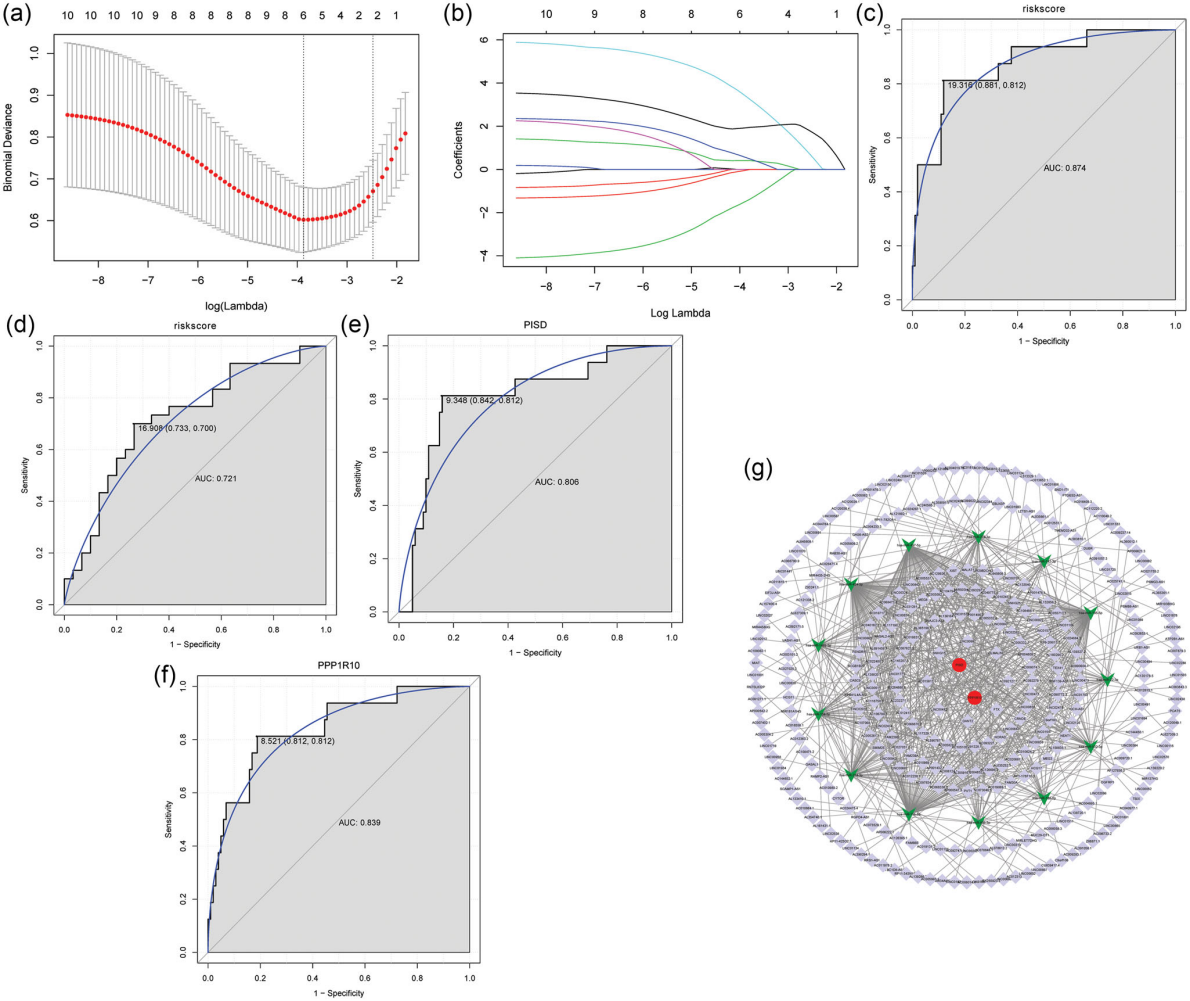
Construction of the diagnostic model: (a) The optimal value of the lambda penalty parameter was determined by 10‐cross validation in GSE60190; (b) LASSO coefficient profiles; (c) ROC analysis of diagnostic model in GSE60190; (d) ROC analysis of diagnostic model in GSE78104; (e) ROC analysis of PISD in GSE60190; (f) ROC analysis of PPP1R10 in GSE60190; and (g) ceRNA regulatory network. Red, green, and purple represents DEmRNAs, miRNAs, and lncRNAs, respectively.

Two signature genes (PISD and PPP1R10) were screened from the GSE60190 dataset (remove GSM1467319 sample) by Lasso method (Figure 5a,b). The risk score formula is as follows: risk score = (0.523 * PISD) + (1.689 * PPP1R10). In the ROC curve analysis, the AUC of risk model was 0.874 (Figure 5c). It is indicated that the risk model may has high diagnostic value in OCD. Validation on the GSE78104 dataset also demonstrated that the model has high diagnostic accuracy (Figure 5d). We also performed ROC analysis for PISD and PPP1R10. The results showed that the AUC of PISD and PPP1R10 were 0.806 and 0.839, respectively (Figure 5e,f). It is indicated that PISD and PPP1R10 may be considered the potential diagnostic gene biomarkers in OCD. To further understand the molecular regulatory mechanisms of PISD and PPP1R10, we constructed a competing endogenous RNA (ceRNA) regulatory network based on the ENCORI (http://starbase.sysu.edu.cn/index.php) database. It was found that 13 miRNAs interacted with PISD and PPP1R10. In addition, 315 lncRNAs interact with 13 miRNAs were also found (Figure 5g).

### 3.6 Multicentric key DEmRNAs

**FIGURE 6:**
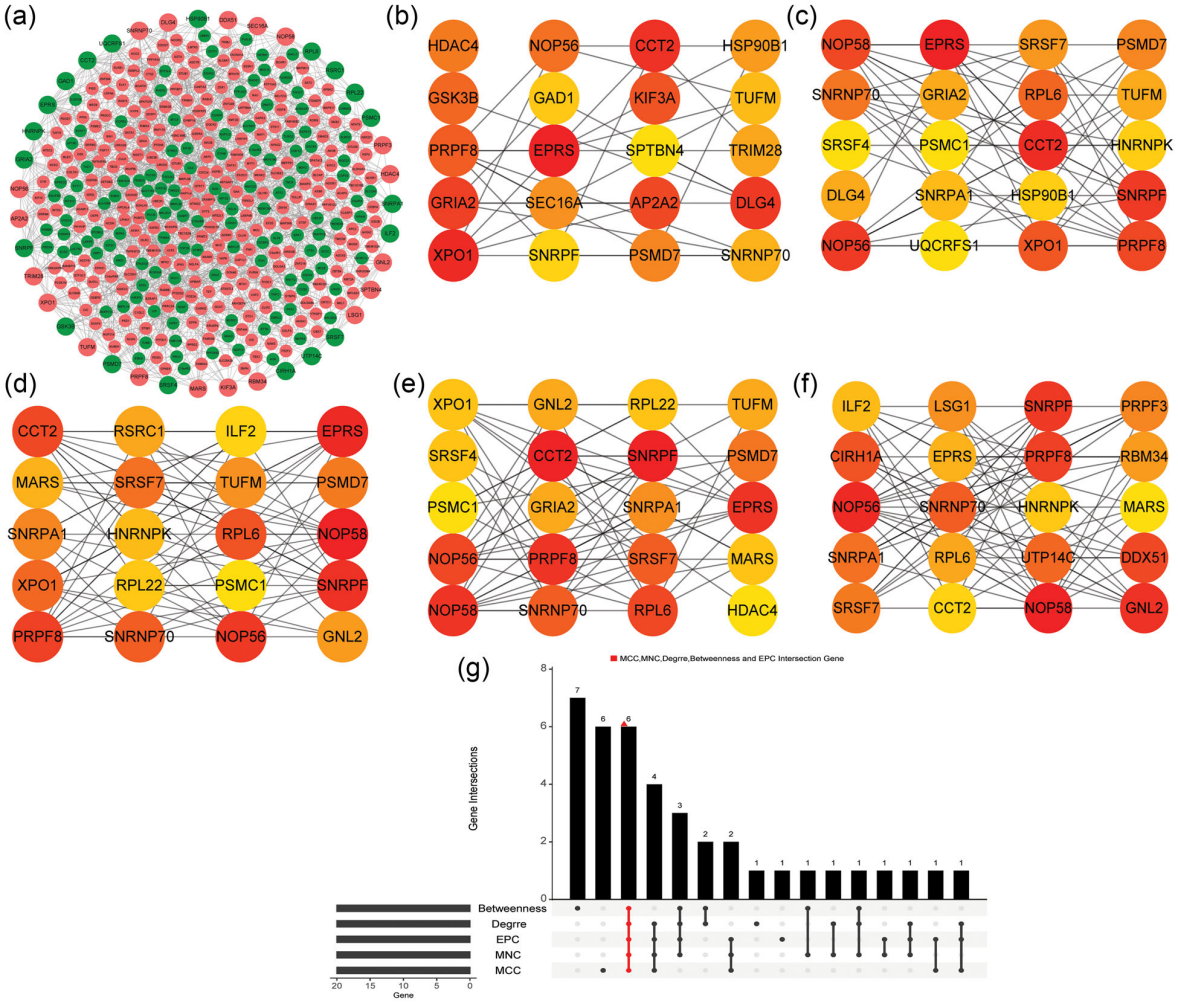
Identification of multicentric key DEmRNAs. (a) PPI network of DEmRNAs. Red and green represents up‐ and downregulated, respectively. (b) PPI network of the top 20 DEmRNAs scored by the betweenness algorithm; (c) PPI network of the top 20 DEmRNAs scored by the degree algorithm; (d) PPI network of the top 20 DEmRNAs scored by the edge percolated component algorithm; (e) PPI network of the top 20 DEmRNAs scored by the maximum neighborhood component algorithm; (f) PPI network of the top 20 DEmRNAs scored by the maximal clique centrality algorithm; and (g) UpSet intersection graph of five groups of algorithms. The darker the color, the greater the gene importance.

**FIGURE 7:**
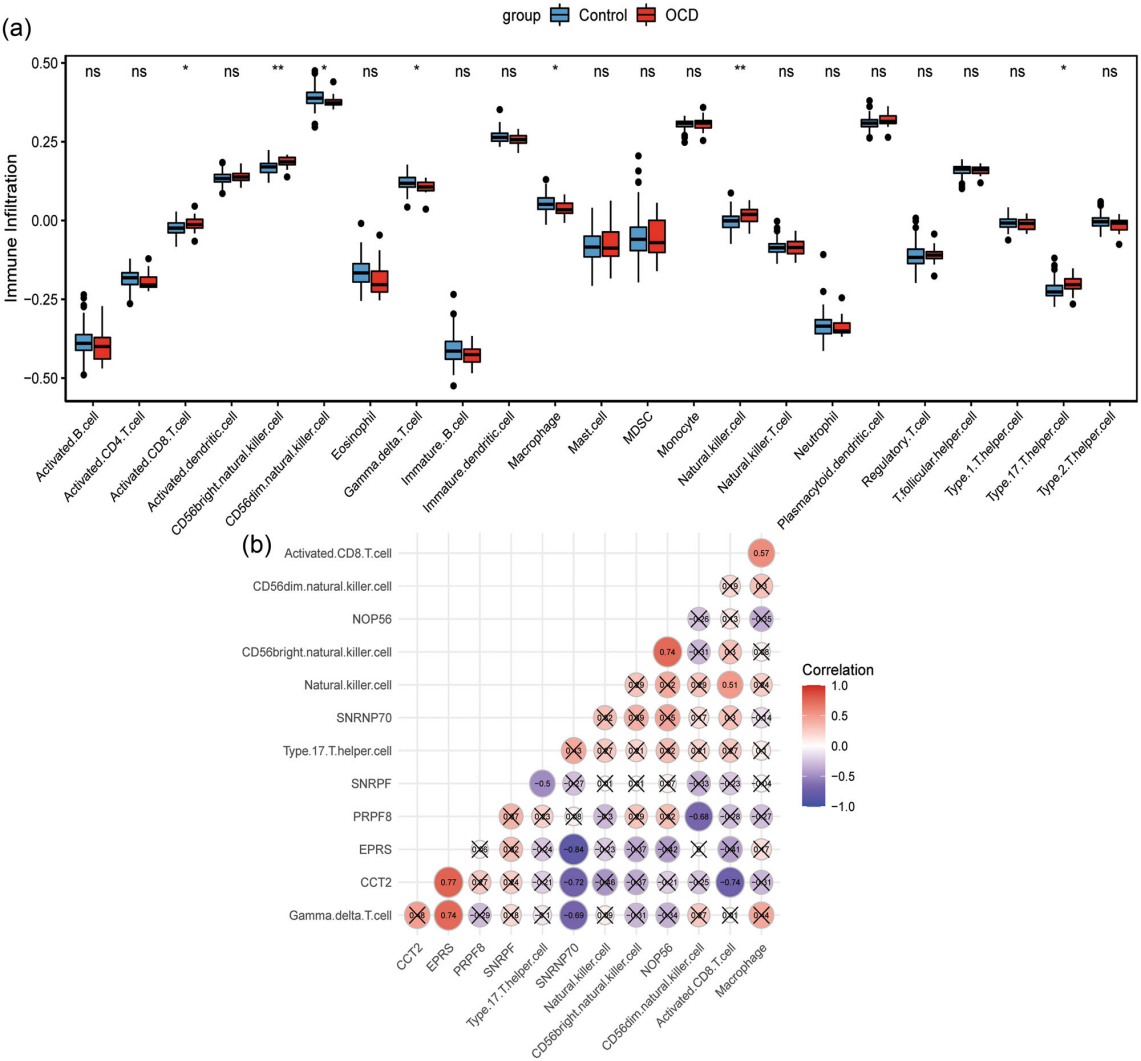
Correlation analysis of immune cells. (a) Differential analysis of immune cell infiltration in OCD and normal control groups. *p < .05; **p < .01; ns represents no statistical significance. (b) Correlation analysis between multicentric key DEmRNAs and differential immune cells. Red and blue represent positive and negative correlations, respectively.

To study the regulatory relationship between DEmRNAs in GSE60190 datasets, we constructed a PPI regulatory network (Figure 6a). Then, five groups of central DEmRNAs were screened by five algorithms in cytoHubba plug‐in (Figure 6b–f). Finally, six multicentric key DEmRNAs (small nuclear ribonucleoprotein polypeptide F [SNRPF], Small nuclear ribonucleoprotein U1 subunit 70 [SNRNP70], pre‐mRNA processing factor 8 [PRPF8], NOP56 ribonucleoprotein [NOP56], glutamyl‐prolyl‐tRNA synthetase [EPRS], and chaperonin containing TCP1 subunit 2 [CCT2]) were screened by UpSet package (Figure 6g). To explore the potential role of immunity in OCD, the levels of immune cell infiltration in the IME of OCD and normal control samples in the GSE60190 dataset (remove GSM1467319 sample) were analyzed. The results showed that Activated.CD8.T.cell, CD56bright.natural.killer.cell, Natural.killer.cell, and Type.17.T.helper.cell had increased infiltration levels in OCD, whereas CD56dim.natural.killer.cell, Gamma.delta.T.cell, and Macrophage had decreased infiltration levels in OCD compared with normal controls (Figure 7a). Subsequently, the Pearson correlation analysis was performed to further explore the correlation between multicentric key DEmRNAs and differential immune cells in OCD. The results showed that CD56bright.natural.killer.cell and NOP56, Gamma.delta.T.cell and EPRS were positively correlated, whereas Gamma.delta.T.cell and SNRNP70, Type.17.T.helper.cell and SNRPF, CD56dim.natural.killer.cell and PRPF8, and Activated.CD8.T.cell and CCT2 were negatively correlated (Figure 7b).

### 3.7 Real‐time‐PCR validation

**FIGURE 8:**
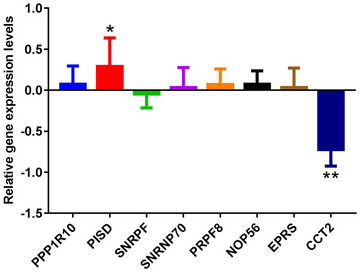
Real‐time‐PCR verification of PPP1R10, PISD, SNRPF, SNRNP70, PRPF8, NOP56, EPRS, and CCT2. *p < .05; **p < .01.

In this study, PPP1R10, PISD, SNRPF, SNRNP70, PRPF8, NOP56, EPRS, and CCT2 were selected for real‐time‐PCR verification. The primers used for real‐time‐PCR verification are shown in Table 1. Real‐time‐PCR validation results showed that compared with the control group, PPP1R10, PISD, SNRNP70, PRPF8, NOP56, and EPRS were upregulated trend, whereas SNRPF and CCT2 were downregulated trend in the OCD group (Figure 8). Among them, PISD and CCT2 have significant differences. In addition, except EPRS, expression trends of other mRNAs were consistent with the bioinformatics analysis. The lack of significance of most mRNAs in real‐time‐PCR results may be caused by the small sample size. Therefore, a large number of samples should be collected for further research.

**TABLE 1:** Primers used for real‐time‐PCR.

Primer name	Primer sequence (5′–3′)
GAPDH‐F (internal reference)	5‐GGAGCGAGATCCCTCCAAAAT‐3
GAPDH‐R (internal reference)	5‐GGCTGTTGTCATACTTCTCATGG‐3
ACTB‐F (internal reference)	5‐CATGTACGTTGCTATCCAGGC‐3
ACTB‐R (internal reference)	5‐CTCCTTAATGTCACGCACGAT‐3
PPP1R10‐F	5‐GTTTTGGGTCCTGGTTGGTT‐3
PPP1R10‐R	5‐CCCATCTCGGTTAAGGAAGC‐3
PISD‐F	5‐CTGCATTCCTGACCGAACCT‐3
PISD‐R	5‐ATGTGGGAACGGAAAGCAGT‐3
SNRPF‐F	5‐GTAGCCTGCAACATTCGGC‐3
SNRPF‐R	5‐CCCTTGTACTCCATTCCCCAC‐3
SNRNP70‐F	5‐CCAAGCTCCGGAGAGAGTTT‐3
SNRNP70‐R	5‐ACCCTCCTGCCATCAATCTT‐3
PRPF8‐F	5‐GATCAAGGTCGAGGTGCAGC‐3
PRPF8‐R	5‐CGTCAGCTGCGATTGTTCCT‐3
NOP56‐F	5‐GCAAATTCCACAGCATCGTT‐3
NOP56‐R	5‐CTGTATTGCGGCACCAATCT‐3
EPRS‐F	5‐GTGTTAACATCCGCGTTAGAGC‐3
EPRS‐R	5‐CTCTGCCTCATTTTCAGCAACT‐3
CCT2‐F	5‐ATATTGCGGGCACAACATTATC‐3
CCT2‐R	5‐ATCTGCCAAACTTCCTCCTAGC‐3

## 4 DISCUSSION

In this study, 10 intersection DEmRNAs (HSPB1, ITPK1, CBX7, PPP1R10, TAOK1, PISD, MKNK2, RWDD1, PPA1, and RELN) were obtained based on WGCNA and gene differential expression analysis. Subsequently, drug prediction was performed on 10 DEmRNAs. However, only four DEmRNAs (HSPB1, TAOK1, MKNK2, and PPA1) predicted related drugs. Previous studies have found that HSPB1, also known as HSP27, plays a key role in neuronal differentiation and neurite growth (Read & Gorman, 2009; Sharp et al., 2013). Pathogenic mutations of HSPB1 are associated with neurodegenerative diseases (Geuens et al., 2017). In addition, polymorphism of HSPB1 is associated with schizophrenia (Kowalczyk & Kucia, 2022). TAOK1 is associated with neurodevelopmental disorder. TAOK1 expression levels affect neural migration in vivo as well as neuronal maturation in vitro (van Woerden & Bos, 2021). Abnormalities in TAOK1 have also been associated with the progression of autism and Alzheimer's disease (Fang et al., 2020). Lowering eIF4E phosphorylation levels by knocking out MKNK1 (also known as MNK1) and MKNK2 (also known as MNK2) genes can trigger behaviors similar to depression and anxiety (Aguilar‐Valles et al., 2018). Up to now, no relevant studies have been found on HSPB1, TAOK1, and MKNK2 in OCD. Inorganic PPA1 is associated with a variety of tumor progression, but no relevant studies have been found in psychiatric and neurological diseases. To our knowledge, this is the first study to show that HSPB1, TAOK1, MKNK2, and PPA1 are differentially expressed in OCD and play a potential regulatory role in OCD progression. In addition, drugs related to HSPB1, TAOK1, MKNK2, and PPA1 were screened out in this study, providing potential research directions for the treatment of OCD.

In order to effectively diagnose OCD, we constructed a diagnostic model. The AUC values of this diagnostic model in the discovery array and validation array were 0.874 and 0.721, respectively. This implies that the diagnostic model has high diagnostic value. The model contains two signature genes (PPP1R10 and PISD). PPP1R10 (also known as CAT53, PNUTS) plays a role in the progression of Alzheimer's disease (Correia et al., 2009; Raha‐Chowdhury et al., 2005). PISD is a mitochondrial disease gene that can cause bone dysplasia and cataracts. However, it has not been found in psychiatric studies. To our knowledge, this is the first study to show that PPP1R10 and PISD are differentially expressed in OCD. In this study, the AUC values for PPP1R10 and PISD were 0.839 and 0.806, respectively. This suggests that PPP1R10 and PISD may be potential biomarkers of OCD. In addition, in order to further understand the molecular mechanisms of PISD and PPP1R10, we constructed a ceRNA regulatory network. It was found that 13 miRNAs interacted with PISD and PPP1R10. A total of 315 lncRNAs interact with 13 miRNAs were also found. This result suggests that the regulatory mechanism of PISD and PPP1R10 is mediated by multiple miRNAs and lncRNAs. Moreover, the construction of ceRNA network provides potential research direction for further exploring the molecular mechanism of OCD.

Six multicentric key DEmRNAs (SNRPF, SNRNP70, PRPF8, NOP56, EPRS, and CCT2) were obtained using the cytoHubba plugin. Previous studies have shown that SNRPF is associated with Alzheimer's disease progression (Zhu et al., 2020). SNRNP70 (also known as SNRP70, U1‐70K) is associated with mental health disorders and is a biomarker of Alzheimer's disease (Camargo et al., 2007; Diner et al., 2014). Expansion of intronic GGCCTG hexanucleotide repeat in NOP56 causes SCA36, a type of spinocerebellar ataxia accompanied by motor neuron involvement (Kobayashi et al., 2011). So far, there is no relevant research on CCT2, EPRS, and PRPF8 in psychiatric diseases. Research in recent years has found that immunity plays a role in the cases and physiology of OCD (Jose et al., 2021; Westwell‐Roper et al., 2022). Therefore, we analyzed the infiltration of immune cells in the OCD IME. The results showed that Activated.CD8.T.cell, CD56bright.natural.killer.cell, Natural.killer.cell, and Type.17.T.helper.cell had increased infiltration levels in OCD, whereas CD56dim.natural.killer.cell, Gamma.delta.T.cell, and Macrophage had decreased infiltration levels in OCD compared with normal controls. Subsequently, to explore the correlation of multicentric key DEmRNAs and differential immune cells, we performed the Pearson correlation analysis. The results showed that CD56bright.natural.killer.cell and NOP56, Gamma.delta.T.cell and EPRS were positively correlated, whereas Gamma.delta.T.cell and SNRNP70, Type.17.T.helper.cell and SNRPF, CD56dim.natural.killer.cell and PRPF8, and Activated.CD8.T.cell and CCT2 were negatively correlated. Our results further elucidate the important role of immunity in OCD. Therefore, we hypothesized that SNRPF, SNRNP70, PRPF8, NOP56, EPRS, and CCT2 may play an important role in the progression of OCD by modulating immune cells. However, the specific molecular mechanisms need to be further studied.

An important signal pathway retrograde endocannabinoid signaling was found in KEGG functional enrichment. The perturbation of the mitochondrial complex underlying the onset of AD is regulated by molecules involved in the retrograde endocannabinoid signaling pathway (Chen et al., 2022). Previous studies have found that retrograde endocannabinoid signaling may be related to the persistent behavior of OCD (Marshall et al., 2018). In this study, retrograde endocannabinoid signaling was found to be a significantly enriched signal notification, which further clarified that retrograde endocannabinoid signaling plays an important regulatory role in OCD. It has laid the theoretical foundation for the future research.

However, there are some limitations in this study. First, the sample size of RT‐PCR verification is too small, and a large number of samples need to be collected for further research. In addition, the specific mechanism of the key molecules obtained in this study in OCD is still unclear, and further research is needed in the future.

In this study, drugs related to HSPB1, TAOK1, MKNK2, and PPA1 were screened out, which provided potential research directions for OCD treatment. A diagnostic model was also constructed based on PPP1R10 and PISD. The AUC value of the diagnostic model implies that the diagnostic model has high diagnostic value. Moreover, six multicentric key DEmRNAs (SNRPF, SNRNP70, PRPF8, NOP56, EPRS, and CCT2) were obtained based on the cytoHubba plugin. Multicentric key DEmRNAs may also play an important role in the progression of OCD by regulating immune cells. In addition, it was found that an important signaling pathway retrograde endocannabinoid signaling may play a regulatory role in OCD. In short, the key molecules obtained in this study lay a foundation for further research on the molecular mechanism of OCD.

## AUTHOR CONTRIBUTIONS

**Xiaoming Zhang**: Conceptualization; investigation; validation; writing—review and editing; writing—original draft. **Yanru Liu**: Data curation; investigation; validation; writing—review and editing. **Bin Guo**: Data curation; investigation. **Binbin Li**: Investigation; resources; software. **Huaqing Liu**: Data curation; validation. **Zhiren Wang**: Conceptualization; project administration; writing—review and editing.

### PEER REVIEW

The peer review history for this article is available at https://publons.com/publon/10.1002/brb3.3412.

## Data Availability

The databases analyzed during the current study are available in the GEO database, and persistent accessible web link to database is https://www.ncbi.nlm.nih.gov/geo/. Accession numbers of the datasets used in the current study are GSE60190 and GSE78104 in GEO. All data generated or analyzed during this study are included in this published article.
